# Transfer-Free Graphene-Like Thin Films on GaN LED Epiwafers Grown by PECVD Using an Ultrathin Pt Catalyst for Transparent Electrode Applications

**DOI:** 10.3390/ma12213533

**Published:** 2019-10-28

**Authors:** Fangzhu Xiong, Weiling Guo, Shiwei Feng, Xuan Li, Zaifa Du, Le Wang, Jun Deng, Jie Sun

**Affiliations:** 1 Key Laboratory of Optoelectronics Technology, College of Microelectronics, Beijing University of Technology, Beijing 100124, China; fangzhuxiong@emails.bjut.edu.cn (F.X.); shwfeng@bjut.edu.cn (S.F.); lixuan@emails.bjut.edu.cn (X.L.); 17801011216@163.com (Z.D.); wangle316@emails.bjut.edu.cn (L.W.); dengsu@bjut.edu.cn (J.D.); 2 Quantum Device Physics Laboratory, Department of Microtechnology and Nanoscience, Chalmers University of Technology, 41296 Gothenburg, Sweden

**Keywords:** transfer-free, PECVD, graphene, gallium nitride, LEDs, transparent electrodes, heat spreading

## Abstract

In this work, we grew transfer-free graphene-like thin films (GLTFs) directly on gallium nitride (GaN)/sapphire light-emitting diode (LED) substrates. Their electrical, optical and thermal properties were studied for transparent electrode applications. Ultrathin platinum (2 nm) was used as the catalyst in the plasma-enhanced chemical vapor deposition (PECVD). The growth parameters were adjusted such that the high temperature exposure of GaN wafers was reduced to its minimum (deposition temperature as low as 600 °C) to ensure the intactness of GaN epilayers. In a comparison study of the Pt-GLTF GaN LED devices and Pt-only LED devices, the former was found to be superior in most aspects, including surface sheet resistance, power consumption, and temperature distribution, but not in optical transmission. This confirmed that the as-developed GLTF-based transparent electrodes had good current spreading, current injection and thermal spreading functionalities. Most importantly, the technique presented herein does not involve any material transfer, rendering a scalable, controllable, reproducible and semiconductor industry-compatible solution for transparent electrodes in GaN-based optoelectronic devices.

## 1. Introduction:

Gallium nitride (GaN) has attracted remarkable attention as an important material for application in optoelectronic and microelectronic devices, such as light-emitting diodes (LEDs), laser diodes (LDs), solar cells (SCs), and high electron mobility transistors (HEMTs) [1,2,3,4]. In order to simultaneously improve the current spreading, current injection and light extraction efficiency, transparent electrodes are commonly used in GaN-based LEDs and SCs. In current optoelectronic devices, the mainstream transparent electrode material is indium tin oxide (ITO) [5]. However, ITO has an increasingly high price as indium is slathered and getting scarce. Also, ITO is nontransparent at very low wavelength regimes and has poor chemical stability, which is not suitable for ultraviolet GaN LEDs [6,7]. Since 2004 [8], graphene has shown great potential in the fields of nanoelectronics, energy, chemistry and biomedicine for its excellent properties, such as high transparency, conductivity, mobility, thermal conductivity and mechanical strength. Graphene is wide-spectrum transparent (from ultraviolet to near infrared) [9] and for every additional layer, the transparency ideally only decreases by 2.3%. In addition, graphene’s preparation process is relatively simple and cheap. As a result, graphene is likely to be a substitute for ITO [10,11]. At present, copper is widely used to catalyze the growth of graphene by chemical vapor deposition (CVD) because copper has low carbon solubility and thus it is easy to form a single layer of graphene. Kim et al. [12] used Cu as a catalyst to grow graphene. At a 372 nm wavelength, two layers of graphene had a transmittance up to 95%, and four layers were up to 89%, better than 372 nm thick ITO’s 68% transmittance at this wavelength range. However, this method requires a process of wet or dry transfer of graphene to the new substrate after growth, which is very complex and often leads to non-ideal interfaces between graphene and gallium nitride, such as metal residues, oxides, holes and wrinkles. Currently, few people have studied graphene on platinum [13,14,15,16,17,18]. According to Gao et al. [13], compared to copper, platinum has a stronger catalytic capacity on hydrocarbon decomposition and subsequent graphene formation. Therefore, in this study, we used ultrathin platinum (2 nm) as a catalyst for direct growth (i.e., transfer-free) of graphene-like thin films (GLTFs) on a GaN/sapphire LED substrate. This method is more reproducible and convenient for industrial production, avoiding a series of problems associated with the transfer process. Indeed, the direct deposition of graphene on the surface of nitride semiconductors is the best strategy to integrate the two types of materials in a controllable manner [19]. Meanwhile, using a plasma-enhanced and vertical cold wall CVD system [20,21,22] not only reduces the growth temperature and protects the material interfaces, it also speeds up the growth, improves the growth efficiency, and reduces the cost as well. Although the quality of a GLTF is yet lower than standard graphene, its properties are good enough to make the GaN LEDs work properly. Through the measurement of electrical, optical, and thermal properties, we found that the addition of GLTFs not only improved the LED luminous current and reduced the turn-on voltage and luminous power consumption, but also had obvious advantages in heat spreading, which has great prospects for improving the reliability, durability and service life of the device. This is one of the key aspects to achieving bright and durable LEDs.

## 2. Experiment

**Figure 1:**
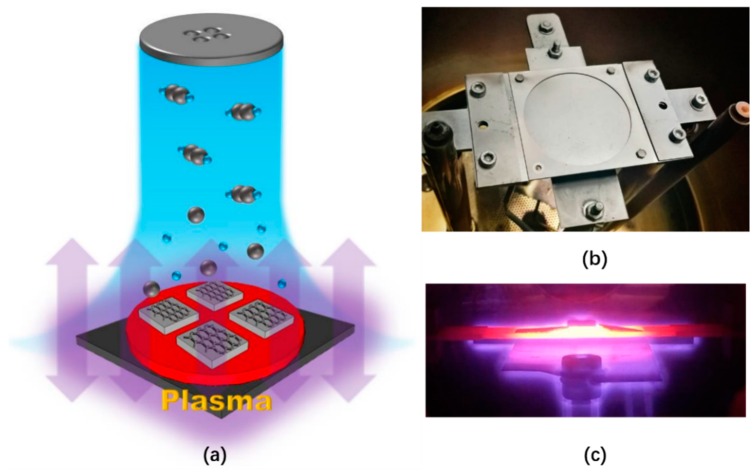
(**a**) The graphene growth schematic. The gas ratio was acetylene:argon = 100:250. We used 40 W-direct current (DC) plasma to assist the growth, which occurred over 25 min at 600 °C and 6 mbar. (**b**) The actual setup of the growth chamber, where the plasma was ignited between the graphitic heater and another graphitic electrode beneath. The round area in the middle of the heater is 2 inches in diameter. (**c**) A photo taken during plasma-enhanced chemical vapor deposition (PECVD), showing the glow map.

**Figure 2:**
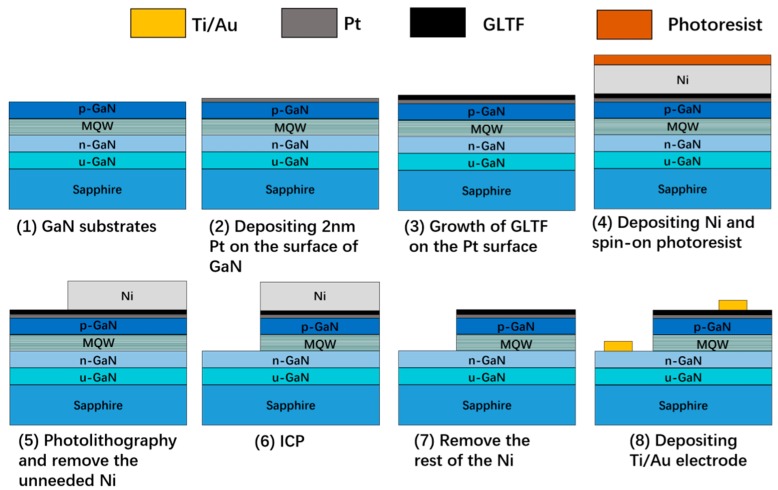
The manufacturing schematic of the light-emitting diodes (LEDs). A device was accomplished by depositing 120 nm of Ni as a dry etching mask on a gallium nitride (GaN) sample pre-deposited with 2 nm Pt and graphene-like thin film (GLTF). Photolithography was then conducted and the Ni patterned. After etching into the heavily doped n-GaN layer using an inductively coupled plasma (ICP) system, the rest of the Ni was removed. Ti/Au (15 nm/300 nm) p and n metal electrodes were also fabricated together by lift-off lithography and sputtering. MQW represents a multiple quantum well.

The plasma-enhanced chemical vapor deposition (PECVD) system used in this experiment was a Black Magic Pro system from AIXTRON Nanoinstruments Ltd. (Swavesey, UK). Unlike the traditional tubular furnace growth, a vertical cold wall CVD system was used here, wherein the only heated area was the middle part of the heater while the rest of the area was “cold”. Faster growth rate and less energy consumption are the main advantages of this system, a schematic of which is shown in Figure 1a. Figure 1b is the actual growth setup. The graphite heater, which was Joule current heated, can support a maximum 2 inch wafer. Another graphite piece was placed just below the heater, and the direct current (DC) plasma was ignited between these two electrodes. However, our growth parameters were not tuned in favor of vertical graphene formation [23], but rather adjusted towards thin film growth. Figure 1c is a photograph taken during the growth phase (side view, the samples on the heater are seen to be immersed in the glowing plasma). The starting samples used in this study were standard commercial GaN LED epitaxial wafers with sapphire substrates. Using 100 sccm acetylene and 250 sccm argon, GLTF grew on the p-GaN (the outermost layer of the LED wafer), which had been pre-deposited with 2 nm platinum (99.99%), and 40 W-DC plasma was used to reduce the growth temperature because of the fact that temperatures above 600 °C tend to result in dense platinum islands on the surface [24,25]. We grew at 600 °C and 6 mbar (chamber pressure) for 25 min to obtain GLTF directly on p-GaN. The graphene deposition mechanism was primarily plasma-enhanced pyrolysis of hydrocarbons, together with some degree of Pt catalysis during the hydrocarbon decomposition and subsequent graphitization. We prepared GaN LEDs on four types of substrates (denoted by samples 1–4), which were: (1) GaN/sapphire LED substrates with no thermal treatment; (2) 2 nm Pt coated GaN/sapphire with no other treatment; (3) 2 nm Pt coated GaN/sapphire annealed at 600 °C for 25 min; and (4) 25 min of GLTF growth at 600 °C on 2 nm Pt coated GaN/sapphire. The individual LED device had a 260 × 515 µm^2^ mesa pattern and was fabricated by two steps of photolithography. The manufacturing schematic of the LED devices is shown in Figure 2. First, 120 nm nickel (99.99%) was deposited on the surface of GLTF as a dry etching mask. After ultraviolet (UV) exposure, high concentration iron trichloride solution was used to remove the unneeded nickel. Afterwards, the wafers with the patterned Ni mask atop were put into an inductively coupled plasma (ICP) dry etching system. The gallium nitride epilayers were etched to a depth of 1.2 μm with a ratio of 8:64 in the SiCl_4_ and Cl_2_ gas mixture to reach the heavily doped n-GaN layer, forming the mesa arrays. The surface GLTF and ultrathin platinum were etched and patterned together. After mesa fabrication, iron trichloride was used to remove the rest of the nickel. Finally, Ti/Au (15 nm/300 nm) p and n metal electrodes were fabricated together by lift-off lithography and sputtering. No annealing was conducted in the metal contacts. For a comparison study, GaN LED devices without GLTF were also fabricated with a similar process (samples 2 and 3).

## 3. Results and Discussion

**Figure 3:**
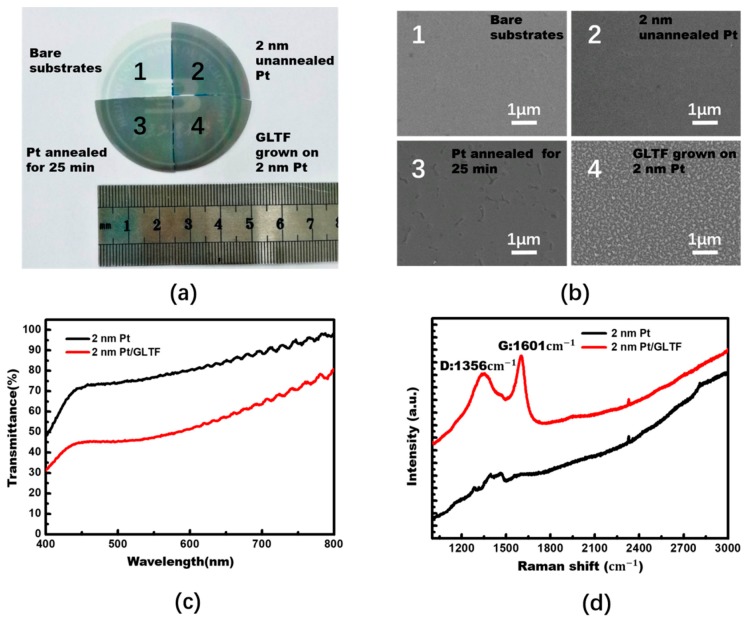
(**a**) Shows GaN LED samples of bare substrates, with 2 nm unannealed platinum, with platinum annealed for 25 min, and with GLTF grown on 2 nm platinum (samples 1–4). (**b**) Shows morphological scanning electron microscope (SEM) images of the four samples. It is clear that the platinum surface roughness increased after annealing. (**c**) Shows the transparencies of the GaN LED epiwafers with 2 nm Pt/graphene (sample 4) and 2 nm Pt (sample 2) in the 400–800 nm range. (**d**) Shows a Raman comparison diagram of samples 2 and 4.

In order to determine whether or not the platinum was affected by the high temperature process, gallium nitride samples with a 2 nm platinum deposit were annealed for 25 min in an argon environment at 600 °C and 40 W-DC plasma. These conditions were nominally the same as the conditions for GLTF growth, except that no carbon source was added. Photographs of the four samples prepared for this study are shown in Figure 3a, labeled as samples 1–4: (1) gallium nitride substrates without any treatment; (2) 2 nm Pt coated p-GaN with no other treatment; (3) 2 nm Pt coated p-GaN in a 600 °C and 40 W DC Ar plasma environment annealed for 25 min; and (4) 25 min of GLTF growth at 600 °C on 2 nm Pt coated p-GaN. For samples 2, 3 and 4, the corresponding sheet resistances were 500, 536 and 472 Ω/sq, respectively. After annealing, the sheet resistance of platinum becomes larger. We noted that the high temperature caused an aggregation effect and, to some extent, turned the platinum film into an island-like structure. Also, the platinum atoms may be partially infiltrated into the p-GaN, further lowering the conductivity of the outer surface. It was clear that the surface roughness of the platinum increased after high temperature annealing. However, after adding the carbon source, the GLTF improved the conductivity of the sample surface despite there still being a high temperature process. Figure 3b shows morphological scanning electron microscope (SEM) images of the four samples. The measured transmittance of GaN LEDs with and without GLTF is plotted as a function of wavelength in Figure 3c. As depicted in the figure, the GaN samples with 2 nm platinum (sample 2) and platinum-GLTF (sample 4) had transparencies in the visible light band of 70–95% and 45–80%, respectively. Both the 2 nm Pt and GLTF reduced the optical transmittance quite a lot. Figure 3d compares the Raman signal of sample 2 and sample 4 at 532 nm wavelength laser excitation. It can be seen that after growing GLTF, two new peaks appear near 1356 cm^−1^ and 1601 cm^−1^, corresponding to the D and G peaks, respectively, which are the signatures of *sp*^2^-C graphitic carbon. The D peak is relatively big, indicating that the material grown in this manner contains a number of defects or disorder, which is a subject of future improvement. The Raman spectrum is not that of standard graphene, but rather a nanographitic structure, and therefore in this paper we term it as GLTF for scientific rigor.

**Figure 4:**
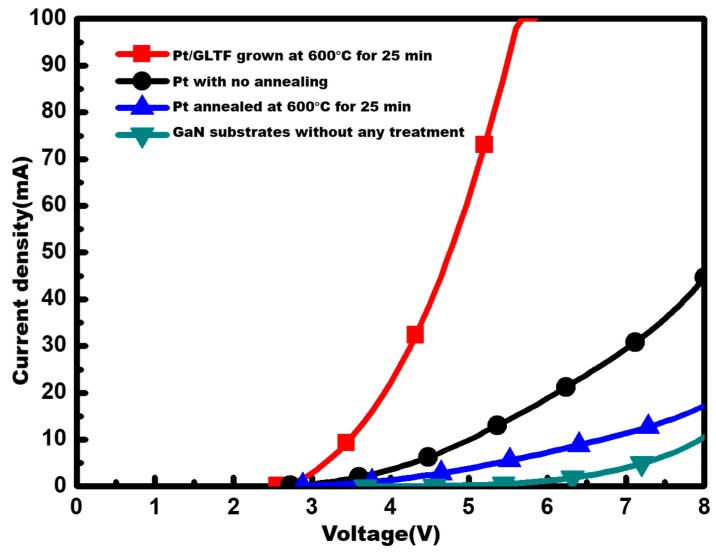
A current–voltage (I–V) curve comparison of the LED devices of GaN substrates without any treatment, with Pt film, with annealed Pt film, and with Pt-GLTF film.

As shown in Figure 4, the current–voltage (I–V) characteristics of the samples—GaN substrates without any treatment (sample 1, green line), with 2 nm Pt film (sample 2, black line), with 2 nm annealed Pt film (sample 3, blue line) and with Pt-GLTF film (sample 4, red line)—were compared after making the LED devices. From this comparison, we suggest that, after annealing at 600 °C with 40 W plasma, the performance of the device with 2 nm Pt was worse than its unannealed counterpart. Even at 8 V, the working current was less than 20 mA. The Pt-GLTF device, on the other hand, had a turn-on voltage as low as 3.8 V, while the turn-on voltages of samples 1–3 were about 5.8 V, 4.2 V and 4.5 V, respectively. At 20 mA, sample four had a forward voltage of 3.9 V, and at 5.6 V the current of the device reached 100 mA, which was the limit of our measurement machine. Hence, after adding the carbon source C_2_H_2_ to form GLTF, the performance of the device appeared to be drastically improved under the same operating conditions. In other words, although the Pt film in this device also underwent a high temperature deteriorating process, the addition of GLTF compensated for this effect and made the device outperform the other two types of devices. In fact, we found the turn-on voltage was strongly affected by the sheet resistance and work function of the transparent electrode materials. In sample 1, which did not have a transparent electrode, it was difficult to inject current uniformly and thus it required a large voltage to turn it on. For sample 2, which had the ultrathin Pt film added, its low sheet resistance and relatively good match to p-GaN’s Fermi level resulted in a reduction of the turn-on voltage. However, after annealing in sample 3, the Pt became islandic and the sheet resistance (hence the turn-on voltage) went up. The turn-on voltage was brought back down by the addition of GLTF in sample 4, as a result of the reduced sheet resistance.

**Figure 5:**
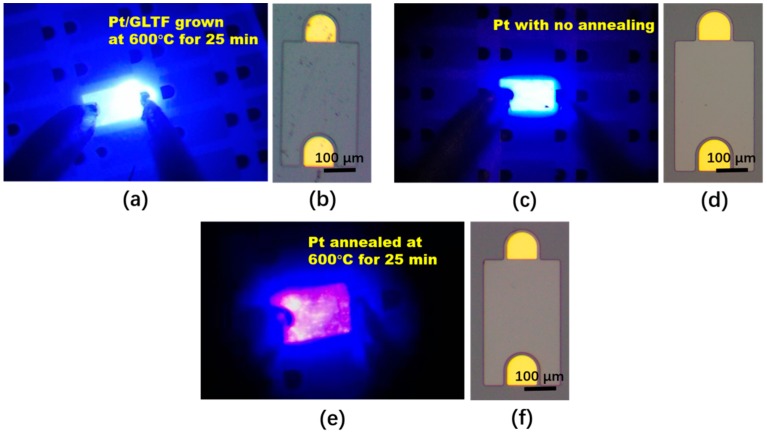
(**a**,**b**) Shows the platinum-GLTF LEDs; (**c**,**d**) shows the 2-nm-platinum-film LEDs; and (**e**,**f**) shows the annealed platinum LEDs.

Figure 5 displays electroluminescent photos of samples 2–4 operating at 5 V, together with their corresponding optical micrographs for a single device. Figure 5a,b shows the platinum-GLTF LEDs (sample 4), Figure 5c,d shows the platinum film LEDs (sample 2), and Figure 5e,f shows the annealed platinum LEDs (sample 3). It can be seen that the Blu-ray luminescence is very uniform for the platinum-GLTF LED and platinum-LED. The current of the annealed platinum LED at the same voltage is not only smaller, but also unevenly distributed, and the surface of the film looks like it has been damaged. It is proved here that the presence of our directly grown GLTF not only has a very good current spreading effect, but also compensates the negative effects from the Pt annealing, making the current and luminous characteristics of the final LEDs better than other devices.

**Figure 6:**
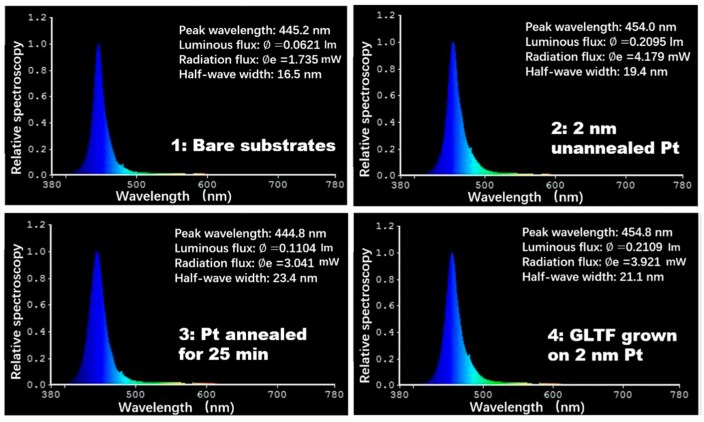
Electroluminescence spectra of GaN LED samples 1–4 measured at 20 mA injection current.

Figure 6 shows the electroluminescence (EL) spectra of samples 1–4 measured at 20 mA current. The blue emissions of samples 2 and 4 are at 454 and 454.8 nm, respectively, and those of samples 1 and 3 show a redshift of around 10 nm. The exact origin is not clear, possibly related to the quantum-confined Stark effect. The luminous and radiation flux values reflect the electrical to optical energy conversion efficiency (wall-plug efficiency). They were low for sample 1 because without any transparent electrodes, the current injection was very poor and only a spot on the mesa emitted light. The situation is much better for samples 2 and 4, but not for sample 3 since the Pt electrode was damaged by the annealing. For a similar reason, the full width at half maximum for samples 2 and 4 is narrower than sample 3. Sample 1 has the narrowest width—not due to good device performance, but because only one spot was emitting light.

**Figure 7:**
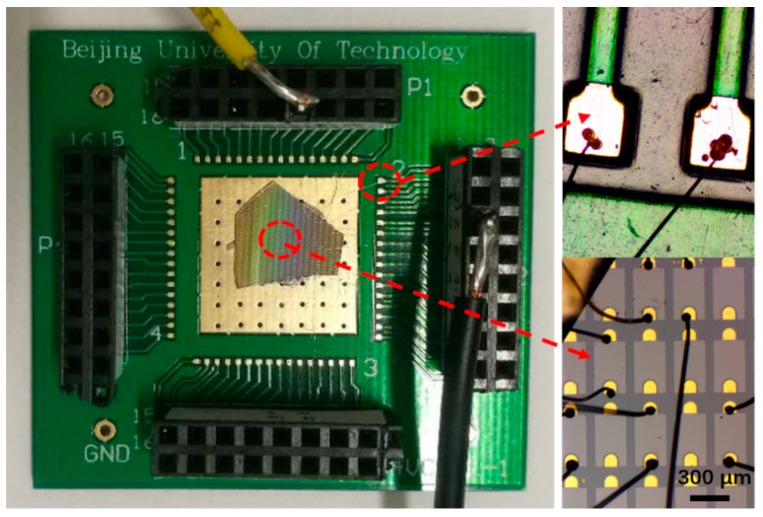
Wire bonding and packaging diagram of the LEDs for thermal measurement. The pentagon in the middle is the GaN piece which was diced to fit the measurement setup.

Finally, in order to study the thermal management characteristics of the as-grown GLTF, we welded and encapsulated three types of LEDs. The package welding diagram of the GaN LEDs is shown in Figure 7. The pentagon in the middle is the GaN piece, the yellow part at the top is the wire, and the red arrows indicate the zoomed-in parts shown in detail in the insets. The p- and n-poles of each LED were connected to the wires via wire bonding for thermal distribution measurements. Using an SC7300M F/2 (MCT) thermal imaging camera (FLIR SYSTEMS, Wilsonville, OR, USA), accurate temperatures of the three LEDs were measured both at room temperature and in operation (in 20 mA constant current mode or 8 V constant voltage mode).

**Figure 8:**
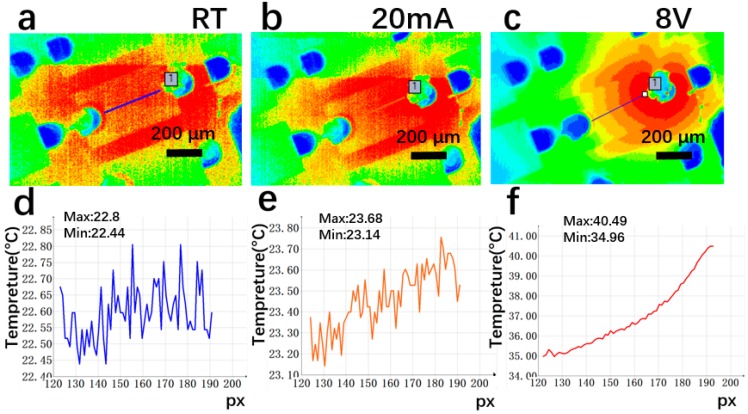
Temperature distribution of platinum-GLTF LEDs (sample 4). In (**b**,**c**), the heat is seen to spread from the p electrode side towards the n electrode side via the GLTF-based transparent electrode. Here, (**a**,**d**) represent room temperature with no operation; (**b**,**e**) represent operation at a constant current of 20 mA; and (**c**,**f**) represent constant voltage working mode at 8 V.

**Figure 9:**
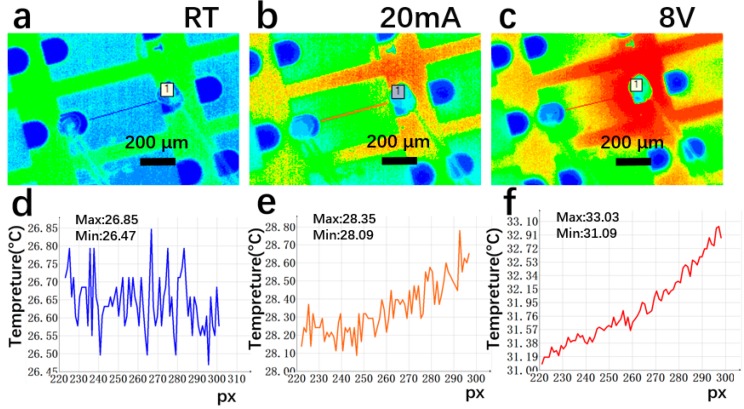
Temperature distribution of platinum film LEDs (sample 2). In (**c**), the heat is accumulated around the p electrode and the nearby trenches and cannot be effectively dissipated or spread. (**a**,**d**): room temperature, no operation; (**b**,**e**): working at constant current 20 mA; (c,**f**): constant voltage (8 V) operation.

**Figure 10:**
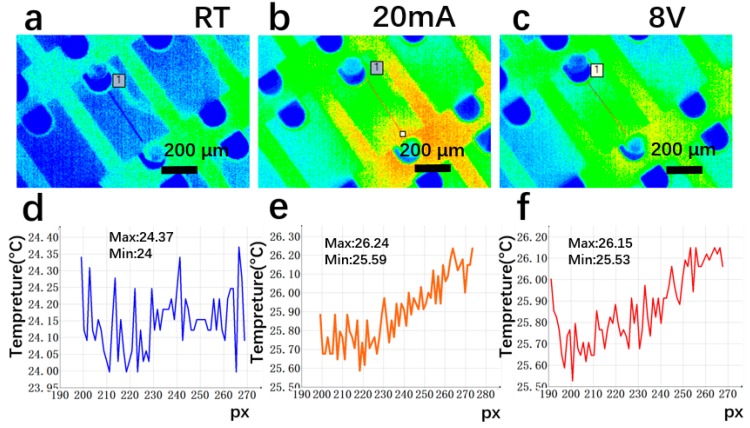
Temperature distribution of platinum-annealed LEDs (sample 3). (**a**,**d**): room temperature; (**b**,**e**): constant current at 20 mA; and (**c**,**f**): constant voltage at 8 V.

Figure 8, Figure 9 and Figure 10 show the platinum-GLTF LEDs (sample 4), platinum film LEDs (sample 2), and platinum-annealed LEDs (sample 3), respectively, under three different conditions for measurement of thermal distribution. In the figures, “px” represents different positions on the film from n to p electrodes along the device (indicated by the solid lines in panels (a–c) in Figure 8, Figure 9 and Figure 10). Panels (a) and (d) were recorded at room temperature when the device was not operating, (b) and (e) were recorded with a constant working current at 20 mA, and (c) and (f) were recorded with a constant voltage at 8 V. Considering Figure 8, by collecting the highest and the lowest temperatures in the temperature distribution along the crossline, it can be estimated that, compared to room temperature (i.e., no operation) at 20 mA, the temperature increased by 0.7–0.88 °C in platinum-GLTF LEDs. At 8 V voltage, platinum-GLTF LEDs reached the limit current of 100 mA in the measurement system, and were about 12.52 to 17.69 °C higher than room temperature.

As shown in Figure 9, the platinum film LED increased its temperature by 1.5–1.62 °C at 20 mA work current, which was much higher than that of the platinum-GLTF LED, indicating that at the same current injection, the Pt film LED generates a lot more heat. Its temperature increased by 4.62–6.18 °C at 8 V work voltage, lower than the Pt-GLTF LED in Figure 8. This, however, is not because it has superior thermal dissipation. Rather, it is because of its low current, caused by the low current injection performance. 

As seen in Figure 10, the temperature of platinum-annealed LEDs increased by 1.59–1.87 °C at 20 mA, while their temperature at 8 V increased by only 1.53–1.78 °C, which may be attributable to the very low current. Based on the data in Figure 8, Figure 9 and Figure 10, we conclude that, at the same current, platinum-GLTF LEDs require less voltage and hence less input power, generating significantly less heat than both nonannealed and annealed platinum LEDs. At the same working voltage, the input power of the Pt-GLTF LEDs is very large because of its large current, which is in turn the result of the good injection performance. Therefore, the temperatures associated with the Pt-GLTF devices are naturally higher than the other two types of devices. Importantly, a heat spreading effect was observed in sample 4. For example, Figure 8c shows the hot spot on the chip is around the p electrode and there is clear spreading of the heat towards the n electrode side across the mesa. A similar effect can be seen in Figure 8b. In contrast, for sample 2 in Figure 9c, the heat is strictly concentrated around the p electrode. Even the trenches between mesas near the p side became hot, but still the heat could not spread towards the n electrode along the mesa. Heat spreading was out of the question for sample 3, as poor injection meant its current was too small to generate any hot spots. Our results show that the heat spreading of GLTF is good, thanks to its very high thermal conductivity.

The overall performance of the devices prepared by our method is reasonably good. However, at this stage, it is still not able to compete with commercial GaN LEDs. For example, the work voltage of standard blue LEDs at 20 mA is only slightly above 3 V. Despite this, compared to graphene-based devices, our devices are very competitive with a 3.9 V work voltage at 20 mA. Commonly, graphene-on-GaN LEDs have forward work voltages of 6–7 V or even more than 10 V [26,27].

## 4. Conclusions

Based on graphene’s high electrical and thermal conductivity, high optical transmittance, rich raw materials, broad spectrum and good chemical stability, there have been a considerable number of papers suggesting graphene is a promising candidate for substitution of ITO transparent electrodes in GaN-based optoelectronics. However, as early as 2012 [28], we recognized the importance of removing the graphene transfer process, because otherwise the technology would conceivably receive no real interest from the semiconductor industry. That is because graphene transfer is tricky, time consuming and irreproducible. In other words, it is incompatible with semiconductor processing, leaving holes, wrinkles and etching residues in the graphene and its interface, as well as damaging the material quality and the graphene-GaN contact. Thus, in this paper, a method of direct growth of GLTF on ultrathin platinum on GaN was demonstrated to avoid transfer. Unlike inert substrates such as SiO_2_, GaN is relatively prone to damage and requires subtle control over the growth process [29,30]. In our case, the growth temperature was reduced by the plasma enhancement technique, and the vertical cold wall system reduced the deposition time. In this way, the exposure of the GaN wafer to high temperatures was limited and the GaN was intact (free from surface decomposition). The 2 nm Pt used in this study helped to catalyze the growth and yield of GLTF, producing results that were better than previous attempts which did not use a catalyst [28], yet was thin enough to let light pass through. Through a comparison study of samples with and without GLTF, we found that, compared to platinum-only LEDs (annealed and nonannealed), the surface sheet resistance and the turn-on voltage of the GLTF devices were smaller. At the same current, the platinum-GLTF LEDs required less voltage and hence less luminous power consumption. In the heat spreading characterization, the GLTF devices were also superior, thanks to the intrinsically high thermal conductivity of GLTF. At 20 mA, the temperature of platinum-GLTF LEDs increased by less than 1 °C, significantly lower than the control samples. Therefore, the as-prepared GLTF-based transparent electrodes had better current spreading, current injection and heat spreading functions in the GaN LEDs. We also identified high temperature annealing as a deteriorating process of 2 nm Pt. However, adding GLTF on top of Pt compensated for this effect and led to better performance. This work confirms that as-grown, transfer-free GLTF has clear advantages in developing high performance, scalable, controllable and reproducible transparent electrodes for GaN-based optoelectronics. At this stage, however, optical transmittance remains a challenge. Seeking an ultrathin metal catalyst with better transparency than Pt and optimization of full-scale growth parameters are expected to refine the technology to a level that is suitable for real industrial applications.
